# Cytotoxic Terpenoids from the Roots of *Dracocephalum taliense*

**DOI:** 10.3390/molecules23010057

**Published:** 2017-12-27

**Authors:** Yanyan Deng, Juan Hua, Wenjia Wang, Zhonglang Zhan, Anqi Wang, Shihong Luo

**Affiliations:** College of Bioscience and Biotechnology, Shenyang Agricultural University, Shenyang 110866, China; hao2yan2hao@126.com (Y.D.); huajuan@mail.kib.ac.cn (J.H.); wenjia9264@126.com (W.W.); zzlomg@126.com (Z.Z.); waq0619@126.com (A.W.)

**Keywords:** *Dracocephalum taliense*, abietane diterpenoid, ursane triterpenoid, cytotoxicity, structural elucidation

## Abstract

A chemical investigation of methanol extract from the roots of *Dracocephalum taliense* led to the isolation of a new aromatic abietane diterpenoid, 12-methoxy-18-hydroxy-sugiol (**1**), and one highly-oxygenated ursane triterpenoid, 2α,3α-dihydroxy-11α,12α-epoxy-urs-28,13β-olide (**2**), together with 15 known natural products (**3**–**17**). Among these, compounds **1**–**13** and **15**–**17** were detected for the first time in the genus of *Dracocephalum*. The structures of all of these isolates were determined by extensively spectroscopic analyses. In the anti-inflammatory assay, compounds **1** and **2** had no obvious inhibitory activity on the release of cytokine IL-2 in lipopolysaccharide-induced RAW 264.7 macrophages. However, compound **2** exhibited significant cytotoxic activity against cell lines HepG2 (IC_50_ = 6.58 ± 0.14 μM) and NCI-H1975 (IC_50_ = 7.17 ± 0.26 μM).

## 1. Introduction

Terpenoids represent the most widespread and largest class of natural products, with more than 80,000 metabolites in a greater family that also includes steroids and carotenoids [1]. Most terpenoids serve a variety of roles in mediating mutualistic and antagonistic interactions among organisms in the natural world [2]. In flowering plants, terpenoids also constitute a large group of naturally-occurring specialized metabolites, especially as common substances in the Lamiaceae family. For example, abundant diterpenoids with diverse structural scaffolds and important pharmaceutical functions have been discovered in the genus *Isodon* [3,4,5]. The genus *Salvia* is a rich source of structurally-diverse abietane and clerodane diterpenoids [6]. A series of uniquely defensive sesterterpenoids have been found in the species of *Leucosceptrum canum* and *Colquhounia coccinea* var. *mollis* by our previous phytochemical investigation [7,8,9,10]. As a result, the chemical and structural diversity of terpenoids and their biological activities are interesting for research. 

**Figure 1:**
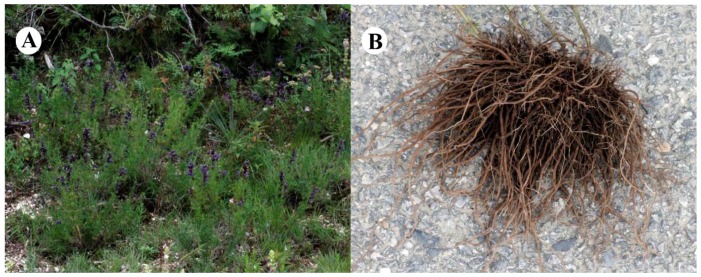
The ecological habitats (**A**) and roots (**B**) of *D. taliense*.

*Dracocephalum* is a herbaceous genus belonging to the Lamiaceae family and is widely distributed in China. Some plants in this genus, including *D. moldavica* and *D. tanguticum*, are broadly used in traditional medicine for gastropathy, tracheitis, and cardiovascular disease in Xinjiang and Tibet [11,12]. Typical metabolites in the plants of this genus were flavonoids and terpenoids [13,14,15,16]. Monoterpenes limonene and α-terpineol might be responsible for antinociceptive properties in the essential oil of *D. kotschyi* [17]. Icetexane diterpenoids, as the main constitutes in *D. komarovi* and *D. kotschyi*, showed moderate trypanocidal activity [16,18,19]. *D. taliense* is a perennial plant with thick cylindric roots and bluish violet flowers on short stalks. It grows in sandy deposits, cliffs, and rocky slopes. It is endemic in distribution in Dali and Shangri-la regions of Yunnan Province (Figure 1). The whole plant of *D. taliense* is used by local people to treat liver disorders, which mainly exhibited good effects on hepatitis and icterus, and also applied for adjusting the stomach [20,21]. However, there is nearly no literature reporting the chemical constituents of this plant. Here, we present the isolation and structural elucidation of the chemical components from the roots of *D. taliense* and their cytotoxic and anti-inflammatory activities.

## 2. Results and Discussion

### 2.1. Structural Elucidation of Compounds

**Figure 2:**
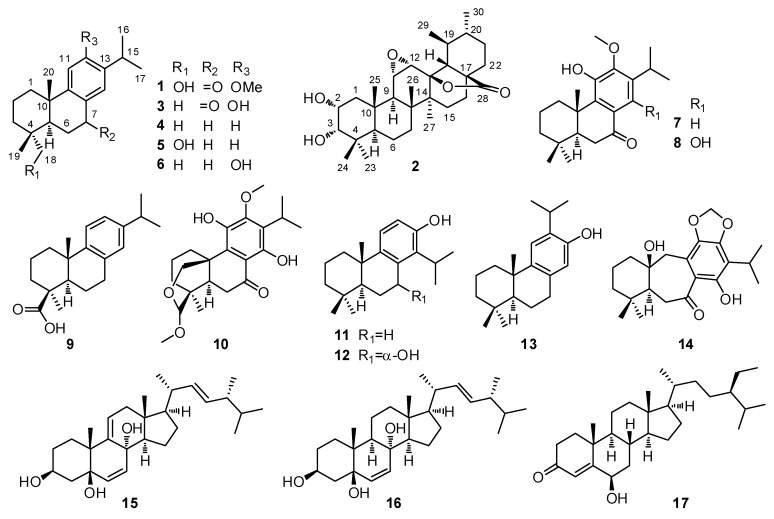
Chemical structures of compounds **1**–**17** from roots of *D. taliense*.

**Figure 3:**
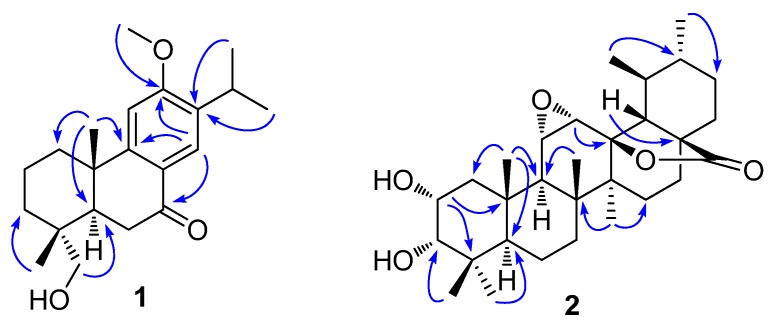
Key HMBC correlations (H→C) of compounds **1** and **2**.

Compound **1** (Figure 2) was obtained as a light yellow oil. Its molecular formula is C_21_H_30_O_3_ according to the ^13^C NMR spectroscopic and high resolution electrospray ionization mass spectroscopy (HRESIMS) data (found: *m*/*z* 353.2092 [M + Na]^+^; calcd. for 353.2087). The ^1^H NMR spectra data of **1** (Table 1, Supplementary Materials Figure S1) exhibited signals for a tetra-substituted benzene ring with protons at *δ*_H_ 6.95 and 7.78, two singlet methyls at *δ*_H_ 1.02 and 1.27, two secondary methyls at *δ*_H_ 1.17 (3H, d, *J* = 7.0 Hz) and 1.19 (3H, d, *J* = 7.0 Hz), one methoxyl signal at *δ*_H_ 3.94, and other signals between 1.04 and 3.24 ppm. The ^13^C NMR and distortionless enhanced polarization transfer (DEPT) spectra (Supplementary Materials Figure S2) demonstrated 21 carbon resonances which were assigned to five methyls, five methylenes (with one oxygenated), four methines, and seven quaternary carbons (with one carbonyl carbon at *δ*_C_ 197.2). These 20 typically skeletal carbons indicated that compound **1** was a characteristic abietane-type diterpenoid [22,23]. In the heteronuclear multiple bond coherence (HMBC) spectrum of **1** (Figure 3), the olefinic proton signal at *δ*_H_ 7.78 (H-14) showed correlations to the carbonyl carbon at *δ*_C_ 197.2 (C-7) and the quaternary carbon at *δ*_C_ 162.3 (C-12), and the methoxyl signal exhibited correlation with the quaternary carbon at *δ*_C_ 162.3, indicating the carbonyl moiety and methoxyl group substituted in C-7 and C-12, respectively. The HMBC correlations of the oxygenated methylenes at *δ*_H_ 3.61 and 3.81 to *δ*_C_ 36.2 (C-3), 39.2 (C-4), 51.1 (C-5), and methyl carbon at *δ*_C_ 27.2 indicated oxygenation of either Me-18 or Me-19. From the 2D rotational nuclear Overhauser effect spectroscopy (ROESY) spectrum of **1**, the correlation between *δ*_H_ 1.27 (Me-20) and 1.02 (Me-19), *δ*_H_ 3.61 (H-18b) and 1.94 (H-5) were observed that confirmed the oxygenation of Me-18. Thus, the chemical structure of **1** was confirmed and named 12-methoxy-18-hydroxy-sugiol. 

Compound **2** was isolated as a white amorphous solid and has a molecular formula of C_30_H_46_O_5_, as determined from its HRESIMS molecular ion at *m*/*z* 509.3234 ([M + Na]^+^; calcd. for 509.3237), accounting to eight double-bond equivalents. The ^1^H NMR spectrum of **2** (Table 1, Supplementary Materials Figure S7) showed seven methyl signals, including two secondary methyls (*δ*_H_ 1.14 (3H, d, *J* = 6.6 Hz) and 0.84 (3H, d, *J* = 7.2 Hz)) and five singlet methyls (*δ*_H_ 1.15, 1.07, 1.05, 1.01, and 0.87), suggesting that compound **2** was an ursane-type triterpenoid [24]. Analysis of ^13^C NMR (Supplementary Materials Figure S8) and heteronuclear single-quantum correlation (HSQC) spectra revealed 30 carbons corresponding to one carbonyl resonance (*δ*_C_ 179.5), six quaternary carbon (with one oxygenated at *δ*_C_ 89.2), nine methines (with four oxygenated), seven methylenes, and seven methyls. Based on the aforementioned ^1^H and ^13^C NMR spectra data indicated that compound **2** was a highly-oxygenated ursane triterpenoid [24]. Comparing the ^13^C NMR data (Table 1) of **2** with those of 2α,3β-dihydroxy-11α,12α-epoxy-urs-28,13β-olide [24] showed that **2** had the same planar structure. The only difference was that the C-3 (*δ*_C_ 83.7) in 2α,3β-dihydroxy-11α,12α-epoxy-urs-28,13β-olide shifted upfield to *δ*_C_ 78.7, accounting the β orientation of 3-OH changed to α in **2**, which was further confirmed by ROESY correlations of H-3 with H-2 and Me-24, Me-25 with H-2 and H-3, and low coupling constant value of ^3^*J*_2-3_ (1.9 Hz). Finally, compound **2** was characterized as shown in Figure 2, and was named as 2α,3α-dihydroxy-11α,12α-epoxy-urs-28,13β-olide.

Twelve known diterpenoids, including sugiol (**3**) [23], abieta-8,11,13-triene (**4**) [25], dehydroabietane (**5**) [26], ferruginol (**6**) [27], cryptojapanol (**7**) [28], inuroyleanol (**8**) [22], callitrisic acid (**9**) [29], 11,14-dihydroxy-12,19-dimethoxy-7-*oxo*-8,11,13-abietatrien-19,20-olide (**10**) [30], totarol (**11**) [31], 7α-hydroxytotarol (**12**) [32], sempervirol (**13**) [33], and cyclocoulterone (**14**) [16], and three steroids, including (22*E*)-ergosta-6,9,22-triene-3β,5β,8α-triol (**15**) [34], (22*E*)-ergosta-6,22-diene-3β,5β,8α-triol (**16**) [34], and stigmast-4-en-6β-ol-3-one (**17**) [35], were also isolated and identified by comparison of their spectroscopic data with the literature.

### 2.2. Bioactivities 

Compounds **1** and **2** were assayed for their cytotoxic activity against different cell lines NCI-H1975, HepG2, and MCF-7, using a previously described MTS (3-(4,5-dimethylthiazol-2-yl)-5(3-carboxymethoxyphenyl)-2-(4-sulfopheny)-2*H*-tetrazolium) method [36]. It was evident that only compound **2** exhibited significant cytotoxic activity, with IC_50_ values of 7.17 ± 0.26 and 6.58 ± 0.14 μM against cell lines NCI-H1975 and HepG2, respectively, which were still less active than the positive control (Table 2). In the anti-inflammatory assay, both of the two compounds did not show obvious inhibitory activity on the release production of cytokine IL-2 in lipopolysaccharide-induced RAW 264.7 macrophages.

Abietane diterpenoids, as a large group of secondary metabolites, exhibited diversely biological properties including antitumour, antituberculostatic, antiplatelet aggregation, and anti-inflammatory activities [28]. 11,14-Dihydroxy-12,19-dimethoxy-7-*oxo*-8,11,13-abietatrien-19,20-olide (**10**) showed inhibition effects against the secretion of LTC_4_ which was more potent than the ketotifen used as a positive control [30]. 7α-Hydroxytotarol (**12**) exhibited cytotoxic activity against wide human cancer cell lines and could also inhibit the growth of Gram-positive bacteria and fungi [32]. The icetexane type diterpenoid, cyclocoulterone (**14**) showed moderate trypanocidal activity against epmastigotes of *Trypanosoma cruzi* [16].

## 3. Experimental

### 3.1. General

Optical rotations were obtained on a Jasco P-1020 spectropolarimeter (Jasco, Tokyo, Japan). UV spectroscopic data were measured on a Shimadzu-210A double-beam spectrophotometer (Shimadzu, Tokyo, Japan). IR spectra of samples in KBr discs were recorded on a Bruker-Tensor-27 spectrometer (Bruker, Karlsruhe, Germany) with KBr pellets. Mass spectra were obtained on an Agilent Q-TOF 6200 spectrometer (Agilent Technologies, Santa Clara, CA, USA). Column chromatographies were performed on 200–300 mesh silica gel (Qingdao Marine Chemical Factory, Qingdao, China), or Sephadex LH-20 (Amersham Phamacia Biotech, Uppsala, Sweden), or MCI gel CHP-20P (75–150 μm, Mitsubishi Chemical Corp., Tokyo, Japan). NMR spectra were measured on a Bruker Avance-600 spectrometer (Bruker, Karlsruhe, Germany) in deuterated solvent with TMS as the internal standard. Semi-preparative HPLC was performed on an Agilent 1260 series instrument (Agilent, Santa Clara, CA, USA) equipped with a quaternary pump, an autosampler, a vacuum degasser, a thermostatted column compartment, a diode array detector and an Eclipse XDB-C_18_ column (5 μm, 9.4 × 250 mm). TLC spots were visualized under UV light and by spraying with 5% H_2_SO_4_ in EtOH, followed by heating.

### 3.2. Plant Material

The roots of *D. taliense* were collected from Shangri-la in Yunnan Province in August 2016 and identified by Dr. Chunlei Xiang. An authentic sample (SYAU-2016-0246) was kept at the College of Bioscience and Biotechnology, Shenyang Agricultural University.

### 3.3. Extraction and Isolation

Dried and powdered roots of *D. taliense* (4.0 kg) were extracted with methanol at room temperature. The crude extract was concentrated in vacuo to obtain 40 g of methanol extract. This fraction was chromatographed on a silica gel column, eluting successively with a solvent gradient system (dichloromethane/acetone, 1:0–0:1) to give six fractions (Frs. A–F). Fr. A (3.7 g) was further subjected to MCI gel column chromatography (methanol/water, 60:40–100:0) to obtain five subfractions (Frs. A1–A5). Fr. A4 (0.25 g) was subjected to column chromatography on silica gel, eluting with petroleum ether/acetone (92:8–0:100) and purified by a Sephadex LH-20 (acetone as eluent) to yield **6** (3.2 mg) and **11** (5.2 mg). Fr. A3 (0.42 g) was chromatographed on Sephadex LH-20 column (acetone as eluent) and then purified by semi-preparative HPLC (methanol/water, 75:25, 3 mL/min) to give **4** (3.0 mg, *t*_R_ 25.9 min), **7** (5.5 mg, *t*_R_ 14.7 min), and **13** (3.5 mg, *t*_R_ 17.1 min). Fr. B (1.5 g) was subjected to column chromatography on silica gel, eluting with petroleum ether/ethyl acetate (85:15) and recrystallized with acetone to yield **17** (55.1 mg).

Fr. C (7.9 g) was further subjected to MCI gel column chromatography (methanol/water, 50:50–100:0) to obtain four subfractions (Frs. C1–C4). Fr. C1 was recrystallized with methanol to yield **2** (7.5 mg). Fr. C2 was subjected to Sephadex LH-20 column (methanol as eluent) and then purified by semi-preparative HPLC (methanol/water, 66:34, 3 mL/min) to give **1** (5.0 mg, *t*_R_ 15.6 min), **3** (3.6 mg, *t*_R_ 17.6 min), **9** (21.7 mg, *t*_R_ 21.5 min), **10** (8.6 mg, *t*_R_ 11.3 min), and **12** (7.8 mg, *t*_R_ 12.7 min). Fr. C2 was subjected to column chromatography on silica gel, eluting with petroleum ether/acetone (7:1) and then purified by semi-preparative HPLC (methanol/water, 70:30, 3 mL/min), to yield **5** (4.5 mg, *t*_R_ 15.9 min), **8** (5.3 mg, *t*_R_ 6.7 min), and **14** (12.5 mg, *t*_R_ 10.4 min). Subfraction Fr. C3 was applied to a silica gel column eluted with petroleum ether/acetone (9:1) and purified by semi-preparative HPLC (methanol/water, 78:22, 3 mL/min) to yield **15** (5.2 mg, *t*_R_ 8.9 min) and **16** (9.8 mg, *t*_R_ 9.6 min).

### 3.4. Spectroscopic Data

12-Methoxy-18-hydroxy-sugiol (**1**): light yellow oil; [α]_D_^25^ = +55.6 (*c* = 0.1, MeOH); UV (MeOH) *λ*_max_ (log *ε*): nm 215 (4.16), 270 (3.88), 364 (2.81); IR (KBr, cm^−1^): *ν*_max_ 3441, 2942, 1637, 1602, 1455, 1375, 1241, 1167, 913; ^1^H and ^13^C NMR data, see Table 1; HRESIMS: *m*/*z*_obsd_ 353.2092 [M + Na]^+^ (*m*/*z*_calcd_ [C_21_H_30_O_3_Na]^+^ = 353.2087).

2α,3α-Dihydroxy-11α,12α-epoxy-urs-28,13β-olide (**2**): white amorphous solid; [α]_D_^25^ = +37.6 (*c* = 0.1, MeOH); UV (MeOH) *λ*_max_ (log *ε*): 202 (3.85) nm; IR (KBr, cm^−1^): *ν*_max_ 3443, 2937, 1778, 1633, 1460, 1390, 1141, 1043; ^1^H and ^13^C NMR data, see Table 1; HRESIMS: *m*/*z*_obsd_ 509.3234 [M + Na]^+^ (*m*/*z*_calcd_ [C_30_H_46_O_5_Na]^+^ = 509.3237).

Sugiol (**3**): light yellow solid; ^13^C NMR (acetone-*d*_6_, 150 MHz) *δ*: 38.6 (t, C-1), 19.6 (t, C-2), 42.1 (t, C-3), 33.8 (s, C-4), 50.5 (d, C-5), 36.4 (t, C-6), 196.9 (s, C-7), 124.4 (s, C-8), 156.9 (s, C-9), 38.6 (s, C-10), 110.4 (d, C-11), 160.7 (s, C-12), 133.7 (s, C-13), 126.4 (d, C-14), 27.4 (d, C-15), 22.8 (q, C-16), 22.6 (q, C-17), 32.9 (q, C-18), 21.6 (q, C-19), 23.5 (q, C-20).

Abieta-8,11,13-triene (**4**), yellow solid; ^13^C NMR (acetone-*d*_6_, 150 MHz) *δ*: 39.7 (t, C-1), 20.0 (t, C-2), 42.4 (t, C-3), 34.0 (s, C-4), 51.5 (d, C-5), 19.8 (t, C-6), 30.5 (t, C-7), 132.6 (s, C-8), 153.0 (s, C-9), 38.1 (s, C-10), 120.9 (d, C-11), 111.3 (d, C-12), 148.8 (s, C-13), 127.1 (d, C-14), 27.4 (d, C-15), 23.0 (q, C-16), 22.9 (q, C-17), 33.6 (q, C-18), 21.9 (q, C-19), 25.2 (q, C-20).

Dehydroabietane (**5**), yellow oil; ^13^C NMR (acetone-*d*_6_, 150 MHz) *δ*: 39.4 (t, C-1), 19.4 (t, C-2), 35.9 (t, C-3), 38.5 (s, C-4), 44.3 (d, C-5), 19.4 (t, C-6), 30.7 (t, C-7), 135.6 (s, C-8), 148.4 (s, C-9), 38.0 (s, C-10), 124.4 (d, C-11), 125.0 (d, C-12), 146.0 (s, C-13), 127.4 (d, C-14), 34.2 (d, C-15), 24.4 (q, C-16), 24.3 (q, C-17), 71.5 (t, C-18), 18.0 (q, C-19), 25.6 (q, C-20).

Ferruginol (**6**), yellow oil; ^13^C NMR (acetone-*d*_6_, 150 MHz) *δ*: 39.7 (t, C-1), 20.0 (t, C-2), 42.4 (t, C-3), 34.0 (s, C-4), 51.5 (d, C-5), 20.0 (t, C-6), 30.5 (t, C-7), 126.3 (s, C-8), 148.8 (s, C-9), 38.2 (s, C-10), 111.3 (d, C-11), 153.1 (s, C-12), 132.6 (s, C-13), 127.1 (d, C-14), 27.4 (d, C-15), 22.9 (q, C-16), 23.0 (q, C-17), 34.0 (q, C-18), 21.9 (q, C-19), 25.2 (q, C-20).

Cryptojapanol (**7**), light yellow solid, ^13^C NMR (methanol-*d*_4_, 150 MHz) *δ*: 37.4 (t, C-1), 20.0 (t, C-2), 42.4 (t, C-3), 34.4 (s, C-4), 52.1 (d, C-5), 36.5 (t, C-6), 201.8 (s, C-7), 129.4 (s, C-8), 140.6 (s, C-9), 41.6 (s, C-10), 149.2 (s, C-11), 152.0 (s, C-12), 140.9 (s, C-13), 117.3 (d, C-14), 27.8 (d, C-15), 23.9 (q, C-16), 23.8 (q, C-17), 33.6 (q, C-18), 21.9 (q, C-19), 17.8 (q, C-20), 61.7 (q, 12-OMe).

Inuroyleanol (**8**), light yellow solid; ^13^C NMR (acetone-*d*_6_, 150 MHz) *δ*: 37.2 (t, C-1), 19.7 (t, C-2), 41.9 (t, C-3), 34.0 (s, C-4), 50.7 (d, C-5), 36.3 (t, C-6), 206.9 (s, C-7), 113.0 (s, C-8), 137.5 (s, C-9), 41.2 (s, C-10), 140.6 (s, C-11), 154.2 (s, C-12), 126.8 (s, C-13), 158.4 (s, C-14), 26.4 (d, C-15), 20.8 (q, C-16), 20.7 (q, C-17), 33.4 (q, C-18), 21.8 (q, C-19), 17.7 (q, C-20), 62.1 (q, 12-OMe).

Callitrisic acid (**9**), yellow solid, ^13^C NMR (methanol-*d*_4_, 150 MHz) *δ*: 38.8 (t, C-1), 21.2 (t, C-2), 40.8 (t, C-3), 44.8 (s, C-4), 54.4 (d, C-5), 22.4 (t, C-6), 33.2 (t, C-7), 136.1 (s, C-8), 146.8 (s, C-9), 39.5 (s, C-10), 126.5 (d, C-11), 124.9 (d, C-12), 146.7 (s, C-13), 127.7 (d, C-14), 34.8 (d, C-15), 24.5 (q, C-16), 24.5 (q, C-17), 181.5 (s, C-18), 29.3 (q, C-19), 23. 8 (q, C-20).

11,14-Dihydroxy-12,19-dimethoxy-7-*oxo*-8,11,13-abietatrien-19,20-olide (**10**), yellow solid; ^13^C NMR (acetone-*d*_6_, 150 MHz) *δ*: 35.5 (t, C-1), 22.4 (t, C-2), 40.1 (t, C-3), 36.8 (s, C-4), 45.1 (d, C-5), 37.9 (t, C-6), 206.0 (s, C-7), 113.7 (s, C-8), 130.1 (s, C-9), 39.8 (s, C-10), 141.8 (s, C-11), 154.2 (s, C-12), 127.7 (s, C-13), 158.0 (s, C-14), 26.4 (d, C-15), 20.7 (q, C-16), 20.7 (q, C-17), 23.4 (q, C-18), 106.0 (d, C-19), 60.0 (t, C-20), 62.1 (q, 12-OMe), 54.9 (q, 19-OMe).

Totarol (**11**), yellow oil; ^13^C NMR (acetone-*d*_6_, 150 MHz) *δ*: 40.4 (t, C-1), 20.2 (t, C-2), 42.3 (t, C-3), 33.8 (s, C-4), 50.7 (d, C-5), 20.1 (t, C-6), 29.5 (t, C-7), 133.8 (s, C-8), 142.4 (s, C-9), 38.3 (s, C-10), 123.5 (d, C-11), 115.0 (d, C-12), 154.2 (s, C-13), 131.3 (s, C-14), 28.1 (d, C-15), 20.5 (q, C-16), 20.5 (q, C-17), 33.6 (q, C-18), 21.9 (q, C-19), 25.6 (q, C-20).

7α-Hydroxytotarol (**12**), yellow oil; ^13^C NMR (acetone-*d*_6_, 150 MHz) *δ*: 43.7 (t, C-1), 20.6 (t, C-2), 43.9 (t, C-3), 34.6 (s, C-4), 53.3 (d, C-5), 40.7 (t, C-6), 65.3 (d, C-7), 131.4 (s, C-8), 141.6 (s, C-9), 38.1 (s, C-10), 124.2 (d, C-11), 115.2 (d, C-12), 154.2 (s, C-13), 131.2 (s, C-14), 28.2 (d, C-15), 20.6 (q, C-16), 20.5 (q, C-17), 34.1 (q, C-18), 23.8 (q, C-19), 27.4 (q, C-20).

Sempervirol (**13**), yellow oil; ^13^C NMR (acetone-*d*_6_, 150 MHz) *δ*: 39.9 (t, C-1), 20.0 (t, C-2), 42.5 (t, C-3), 33.9 (s, C-4), 51.5 (d, C-5), 19.9 (t, C-6), 30.8 (t, C-7), 133.7 (s, C-8), 141.9 (s, C-9), 38.1 (s, C-10), 122.7 (d, C-11), 132.8 (s, C-12), 152.6 (s, C-13), 115.3 (d, C-14), 27.9 (d, C-15), 23.1 (q, C-16), 23.0 (q, C-17), 33.7 (q, C-18), 21.9 (q, C-19), 25.4 (q, C-20).

Cyclocoulterone (**14**), light yellow solid, ^13^C NMR (acetone-*d*_6_, 150 MHz) *δ*: 39.3 (t, C-1), 19.6 (t, C-2), 41.8 (t, C-3), 34.7 (s, C-4), 50.8 (d, C-5), 40.7 (t, C-6), 209.9 (s, C-7), 113.2 (s, C-8), 116.7 (s, C-9), 73.2 (s, C-10), 140.1 (s, C-11), 151.5 (s, C-12), 117.0 (s, C-13), 161.2 (s, C-14), 25.1 (d, C-15), 21.0 (q, C-16), 20.9 (q, C-17), 32.3 (q, C-18), 21.8 (q, C-19), 42.2 (t, C-20), 101.9 (t, –OCH_2_O–).

(22*E*)-Ergosta-6,9,22-triene-3β,5β,8α-triol (**15**), white solid, ^13^C NMR (CDCl_3_, 150 MHz) *δ*: 32.6 (t, C-1), 30.6 (t, C-2), 66.3 (d, C-3), 36.1 (t, C-4), 82.7 (s, C-5), 135.4 (d, C-6), 130.7 (d, C-7), 78.3 (s, C-8), 142.5 (s, C-9), 37.9 (s, C-10), 119.7 (d, C-11), 41.2 (t, C-12), 43.6 (s, C-13), 48.1 (d, C-14), 20.9 (t, C-15), 28.6 (t, C-16), 55.8 (d, C-17), 13.0 (q, C-18), 25.5 (q, C-19), 39.9 (d, C-20), 20.7 (q, C-21), 135.1 (d, C-22), 132.4 (d, C-23), 43.2 (d, C-24), 33.0 (d, C-25), 19.6 (q, C-26), 19.9 (q, C-27), 17.5 (q, C-28).

(22*E*)-Ergosta-6,22-diene-3β,5β,8α-triol (**16**), white solid, ^13^C NMR (CDCl_3_, 150 MHz) *δ*: 34.7 (t, C-1), 30.1 (t, C-2), 66.4 (d, C-3), 36.9 (t, C-4), 82.1 (s, C-5), 130.7 (d, C-6), 135.4 (d, C-7), 79.4 (s, C-8), 51.0 (d, C-9), 36.9 (s, C-10), 20.6 (t, C-11), 39.3 (t, C-12), 44.5 (s, C-13), 51.7 (d, C-14), 28.6 (t, C-15), 23.4 (t, C-16), 56.2 (d, C-17), 12.8 (q, C-18), 18.2 (q, C-19), 39.7 (d, C-20), 19.6 (q, C-21), 132.3 (d, C-22), 135.2 (d, C-23), 42.7 (d, C-24), 33.0 (d, C-25), 19.9 (q, C-26), 17.5 (q, C-27), 20.9 (q, C-28).

Stigmast-4-en-6β-ol-3-one (**17**), colorless oil, ^13^C NMR (acetone-*d*_6_, 150 MHz) *δ*: 38.0 (t, C-1), 34.8 (t, C-2), 199.4 (s, C-3), 126.3 (d, C-4), 169.2 (s, C-5), 73.4 (d, C-6), 39.9 (t, C-7), 30.6 (d, C-8), 54.7 (d, C-9), 38.7 (s, C-10), 21.7 (t, C-11), 40.5 (t, C-12), 43.2 (s, C-13), 56.7 (d, C-14), 24.8 (t, C-15), 28.9 (t, C-16), 56.9 (d, C-17), 12.2 (q, C-18), 19.6 (q, C-19), 36.9 (d, C-20), 19.3 (q, C-21), 34.6 (t, C-22), 26.7 (t, C-23), 46.7 (d, C-24), 29.9 (d, C-25), 20.1 (q, C-26), 19.1 (q, C-27), 23.7 (t, C-28), 12.3 (q, C-29).

### 3.5. Cytotoxic Assay

The in vitro cytotoxic activity of compounds **1** and **2** were determined by the MTS method [36]. The tested human cancer cell lines, including human lung adenocarcinoma cell line (NCI-H1975), human hepatocellular carcinoma cell line (HepG2), and human breast adenocarcinoma cell line (MCF-7), were seeded in 96-well plates, and then the plates were incubated for 24 h at 37 °C in a 5% CO_2_ incubator. Subsequently, the compounds were added at a dosage of 0, 0.128, 0.256, 0.512, 1, 2, 5, 10, 20, 40, and 80 μM. After 72 h, MTS was added to the culture medium and the absorbance at 490 nm using a microplate reader (Bio-Rad, Hercules, CA, USA). Each sample was carried out in triplicate. The evaluation of IC_50_ values were calculated with the GraphPad Prism 5.01 (GraphPad Software Inc., San Diego, CA, USA) software. 

### 3.6. Anti-Inflammatory Assay

The murine macrophage RAW 264.7 cell line was cultured in DMEM medium supplemented with 10% heated-inactivated fetal bovine serum in a 37 °C, 5% CO_2_ incubator. Before the anti-inflammatory assay, test compounds were assessed for their cytotoxicity against the RAW 264.7 cell line and were found to be non-toxic at the tested concentrations (40, 20, 10, 5, and 0 μM). Anti-inflammatory activity was assessed by enzyme-linked immunosorbent assay (ELISA, BD Biosciences, Mountain View, CA, USA) using commercial interleukin-2 (IL-2) detecting kits as previously described [37].

### 3.7. Statistical Analysis

The results were presented as mean values ± SD (standard deviations) of the three replicates.

## 4. Conclusions

Previous phytochemical investigation has revealed that flavonoids and terpenoids were the major constituents of genus *Dracocephalum* [11]. Biological studies on these flavonoids have revealed their broad pharmacological activities, especially antioxidant, immunomodulatory, and cytotoxic activities, as well as cardiovascular protective effects [11,13,14,15,38,39]. Trypanocidal diterpenoids with icetexane and octahydroindene skeletons were discovered from the whole plants of *D. komarovi* [16,19]. In this work, a new aromatic abietane diterpenoid, and one highly-oxygenated ursane triterpenoid, together with 15 known compounds, belonging to abietane (**3**–**13**) and icetexane (**14**) diterpenoids, and steroids (**15**–**17**), were isolated from the roots of *D. taliense*. Based on literature reviews, all the chemical constituents, except compound **14**, were detected for the first time in the genus of *Dracocephalum*. Although, compounds **1** and **2** were inactive on the release of cytokine IL-2 in lipopolysaccharide-induced RAW 264.7 macrophages, compound **2** showed significant cytotoxic activity against cell lines HepG2 and NCI-H1975. The results increase the chemical diversity and bioactive constituents of secondary metabolites produced by *D. taliense*.

## Figures and Tables

**Table 1:** ^1^H (600 MHz) and ^13^C (150 MHz) NMR spectral data of compounds **1** and **2**.

Position	1 *^a^*		2 *^b^*	
	*δ*_H_ (ppm), (Hz)	*δ*_C_ (ppm)	*δ*_H_ (ppm), (Hz)	*δ*_C_ (ppm)
1a	2.44, m	38.8, t	1.91, m	41.5, t
1b	1.55, m		1.48, m	
2a	1.79, m	19.4, t	4.07, m	66.0, d
2b	1.61, m			
3a	1.96, m	36.2, t	3.46, d (1.9)	78.7, d
3b	1.04, m			
4	-	39.2, s	-	38.2, s
5	1.94, m	51.1, d	1.20, m	47.4, d
6a	2.70, m	36.5, t	1.47, m (2H)	17.3, t
6b	2.64, m			
7a	-	197.2, s	1.29, m	31.2, t
7b			1.14, m	
8	-	124.7, s	-	41.6, s
9	-	157.3, s	1.70, d (1.5)	51.0, d
10	-	39.0, s	-	37.7, s
11	6.95, s	106.0, d	3.14, dd (1.5, 3.7)	54.4, d
12	-	162.3, s	2.94, d (3.7)	56.2, d
13	-	135.4, s	-	89.2, s
14	7.78, s	125.5, d	-	41.2, s
15a	3.24, m	27.1, d	1.71, m	26.7, t
15b			1.54, m	
16a	1.17, d (3H, 7.0)	22.7, q	2.22, m	22.4, t
16b			1.31, m	
17	1.19, d (3H, 7.0)	22.8, q	-	45.0, s
18a	3.81, d (10.7)	64.7, t	1.92, m	53.9, d
18b	3.61, d (10.7)			
19	1.02, s (3H)	27.2, q	2.30, m	32.9, d
20	1.27, s (3H)	23.9, q	1.87, m	34.6, d
21a			1.51, m	28.2, t
21b			1.05, m	
22a			1.68, m	25.5, t
22b			1.58, m	
23			1.01, s (3H)	28.2, q
24			0.87, s (3H)	21.3, q
25			1.07, s (3H)	18.5, q
26			1.05, s (3H)	20.3, q
27			1.15, s (3H)	16.0, q
28			-	179.5, s
29			1.14, d (3H, 6.6)	18.2, q
30			0.84, d (3H, 7.2)	11.1, q
12-OMe	3.94, s (3H)	56.0, q		

*^a^* recorded in acetone-*d*_6_; *^b^* recorded in CDCl_3_.

**Table 2:** Cytotoxicity and Anti-inflammatory activity of compounds from *D. taliense*.

No	Cytotoxicity to Different Cell Lines/IC_50_ (μM)			Inhibitory Activity on Inflammatory Cytokine (μM)
	NCI-H1975	HepG2	MCF-7	IL-2
**1**	>80	>80	>80	>40
**2**	7.17 ± 0.26	6.58 ± 0.14	>80	>5
PC *^a^*	(6.82 ± 0.24) × 10^−3^	(34.72 ± 2.31) × 10^−3^	(54.35 ± 7.72) × 10^−3^	(2.38 ± 0.28) × 10^−2^

*^a^* Taxol was used as a positive control (PC) for cytotoxic assay; cyclosporine A was used as a positive control for anti-inflammatory assay. NCI-H1975, human lung adenocarcinoma cell line; HepG2, human hepatocellular carcinoma cell line; MCF-7, human breast adenocarcinoma cell line. Values are mean ± SD (*n* = 3).

## Supplementary Materials

Supplementary materials are available online. NMR spectra data (Figures S1–S12) of compounds **1** and **2** are available in the Supplementary Material.
