# Supplementary material for: Cytotoxic Terpenoids from the Roots of Dracocephalum taliense
Source: Molecules. 2017 Dec 27;23(1):57. doi: 10.3390/molecules23010057 (PMC6017833; doi:10.3390/molecules23010057)
Supplement: Supplementary file 1 [file molecules-23-00057-s001.docx]

**Supplementary materials for**

Cytotoxic Terpenoids from the Roots of *Dracocephalum taliense*

Yanyan Deng, Juan Hua, Wenjia Wang, Zhonglang Zhan, Anqi Wang and Shihong Luo *

College of Bioscience and Biotechnology, Shenyang Agricultural University, Shenyang 110866, China; [hao2yan2hao@126.com](mailto:hao2yan2hao@126.com) (Y.D.); huajuan@mail.kib.ac.cn (J.H.); [wenjia9264@126.com](mailto:wenjia9264@126.com) (W.W.);
[zzlomg@126.com](mailto:zzlomg@126.com) (Z.Z.); [waq0619@126.com](mailto:waq0619@126.com) (A.W.)

***** Correspondence: luoshihong@syau.edu.cn; Tel.: +86-24-8848-7163

**Contents**

**Spectra data of compounds 1 and 2.**

**Figure S1.** ^1^H NMR spectrum of compound **1** recorded at 600 MHz in acetone-*d*_6_.

**Figure S2.** ^13^C NMR and DEPT spectra of compound **1** recorded at 150 MHz in acetone-*d*_6_.

**Figure S3.** HSQC spectrum of compound **1**.

**Figure S4.** HMBC spectrum of compound **1**.

**Figure S5.** ^1^H-^1^H COSY spectrum of compound **1**.

**Figure S6.** ROESY spectrum of compound **1**.

**Figure S7.** ^1^H NMR spectrum of compound **2** recorded at 600 MHz in CDCl_3_.

**Figure S8.** ^13^C NMR and DEPT spectra of compound **2** recorded at 150 MHz in CDCl_3_.

**Figure S9.** HSQC spectrum of compound **2**.

**Figure S10.** HMBC spectrum of compound **2**.

**Figure S11.** ^1^H-^1^H COSY spectrum of compound **2**.

**Figure S12.** ROESY spectrum of compound **2**.

**
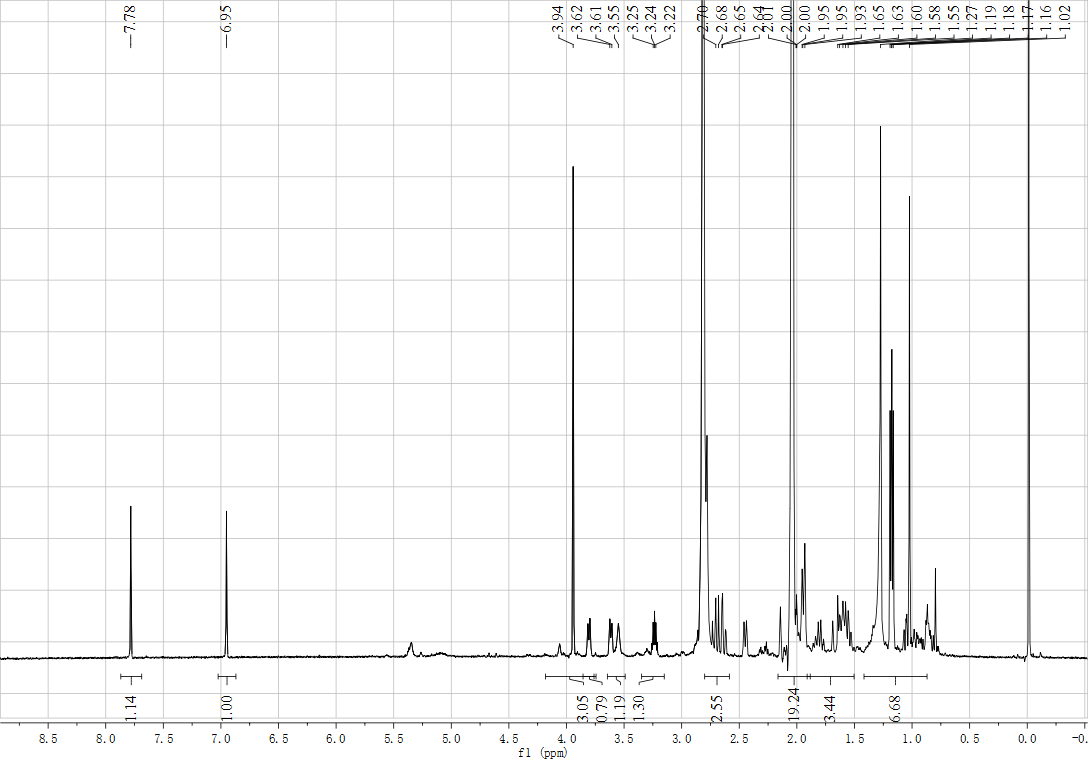
**

**Figure S1.** ^1^H NMR spectrum of compound **1** recorded at 600 MHz in acetone-*d*_6_.

**
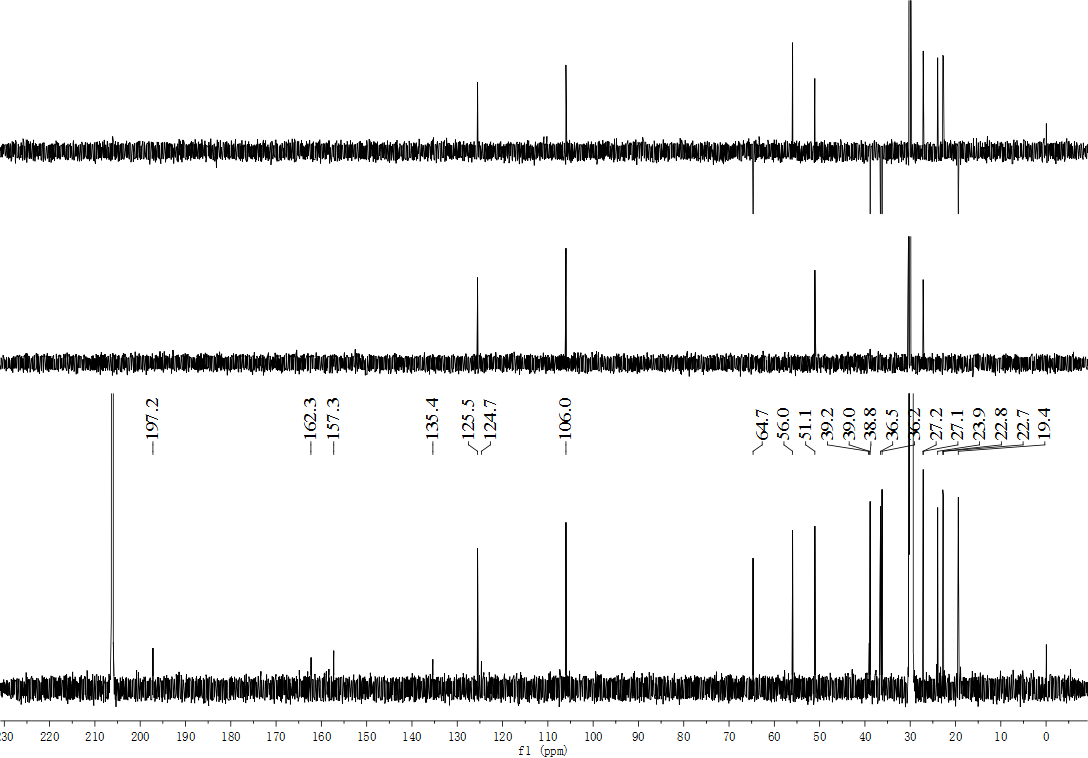
**

**Figure S2.** ^13^C NMR and DEPT spectra of compound **1** recorded at 150 MHz in acetone-*d*_6_.

**
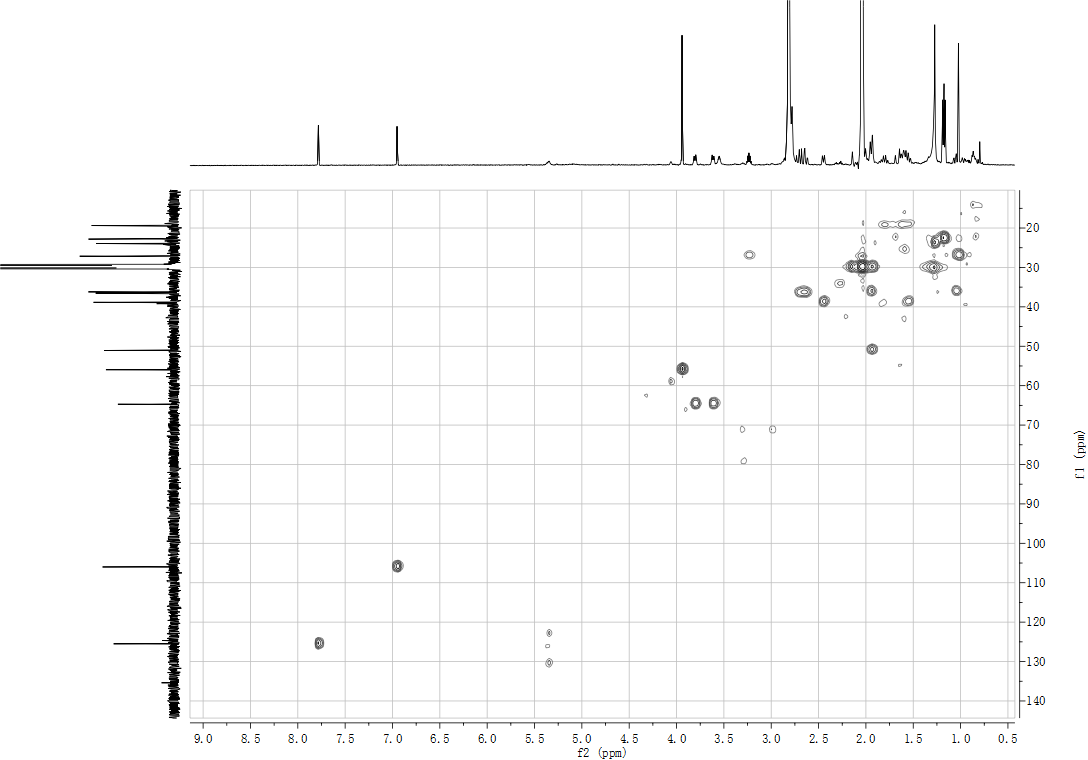
**

**Figure S3.** HSQC spectrum of compound **1**.

**
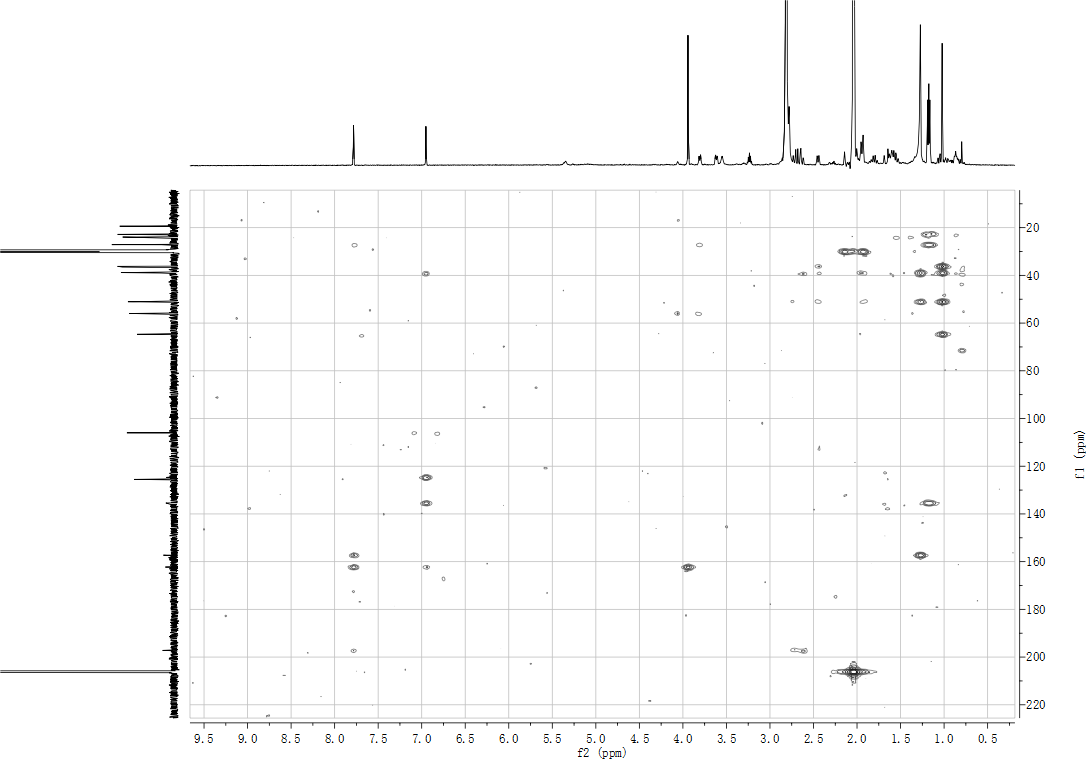
**

**Figure S4.** HMBC spectrum of compound **1**.

**
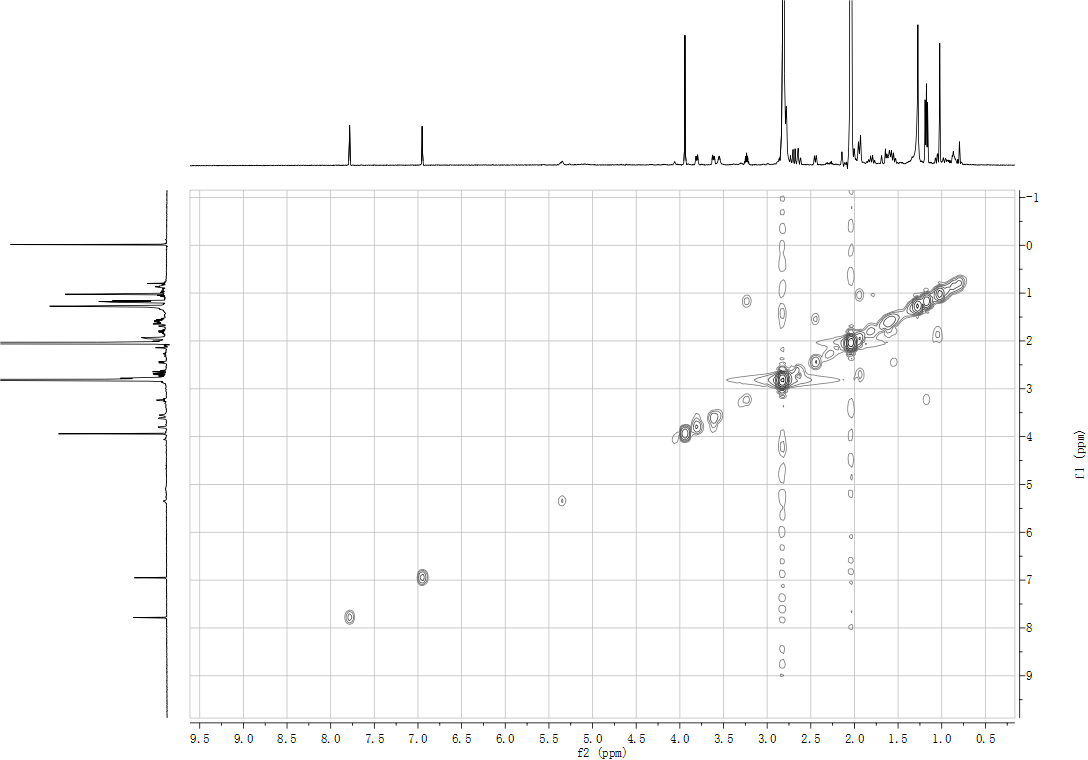
**

**Figure S5.** ^1^H-^1^H COSY spectrum of compound **1**.

**
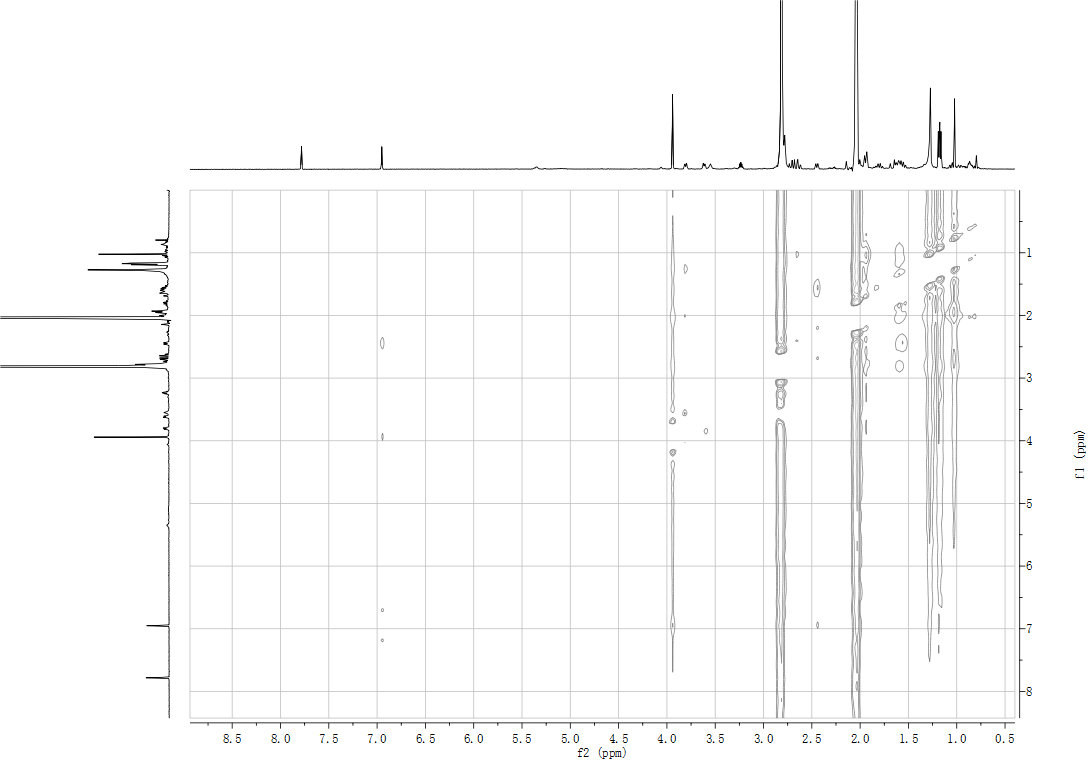
**

**Figure S6.** ROESY spectrum of compound **1**.

**
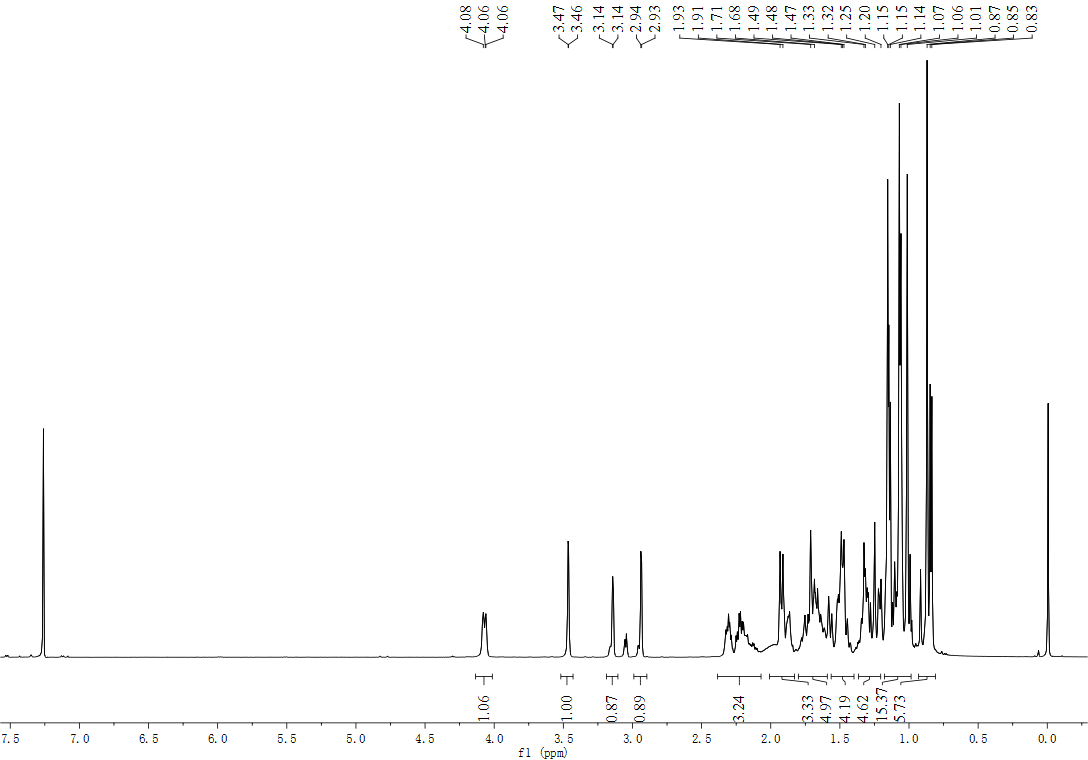
**

**Figure S7.** ^1^H NMR spectrum of compound **2** recorded at 600 MHz in CDCl_3_.

**
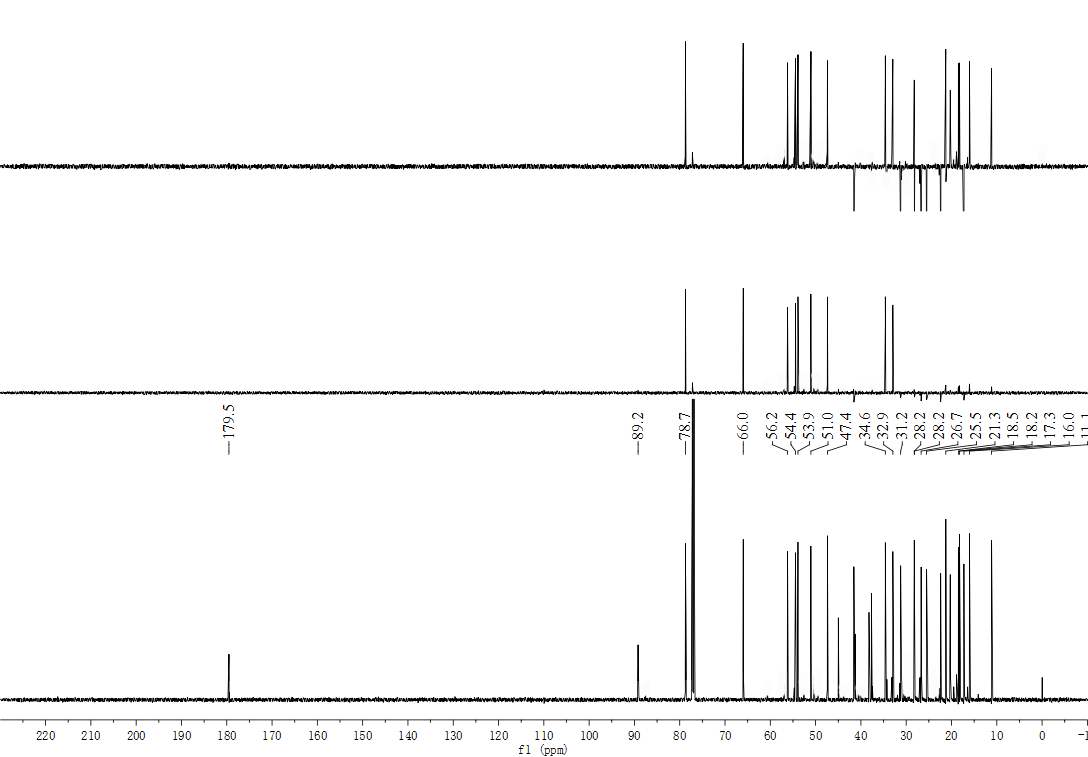
**

**Figure S8.** ^13^C NMR and DEPT spectra of compound **2** recorded at 150 MHz in CDCl_3_.


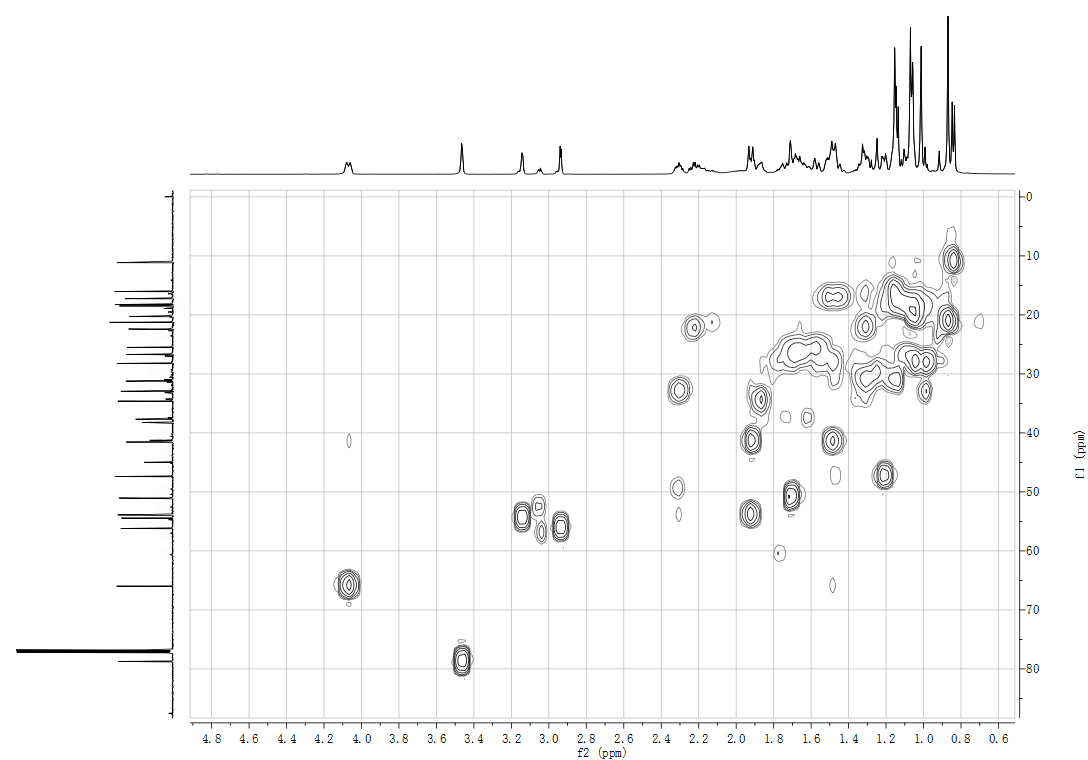


**Figure S9.** HSQC spectrum of compound **2**.

**
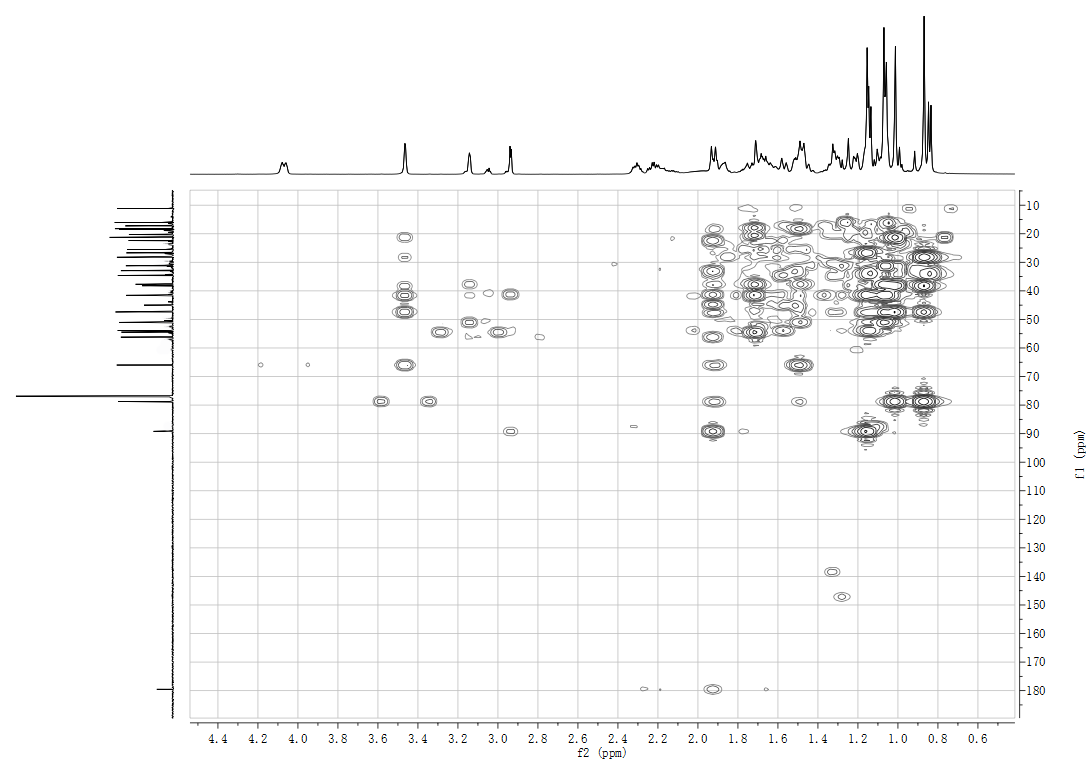
**

**Figure S10.** HMBC spectrum of compound **2**.

**
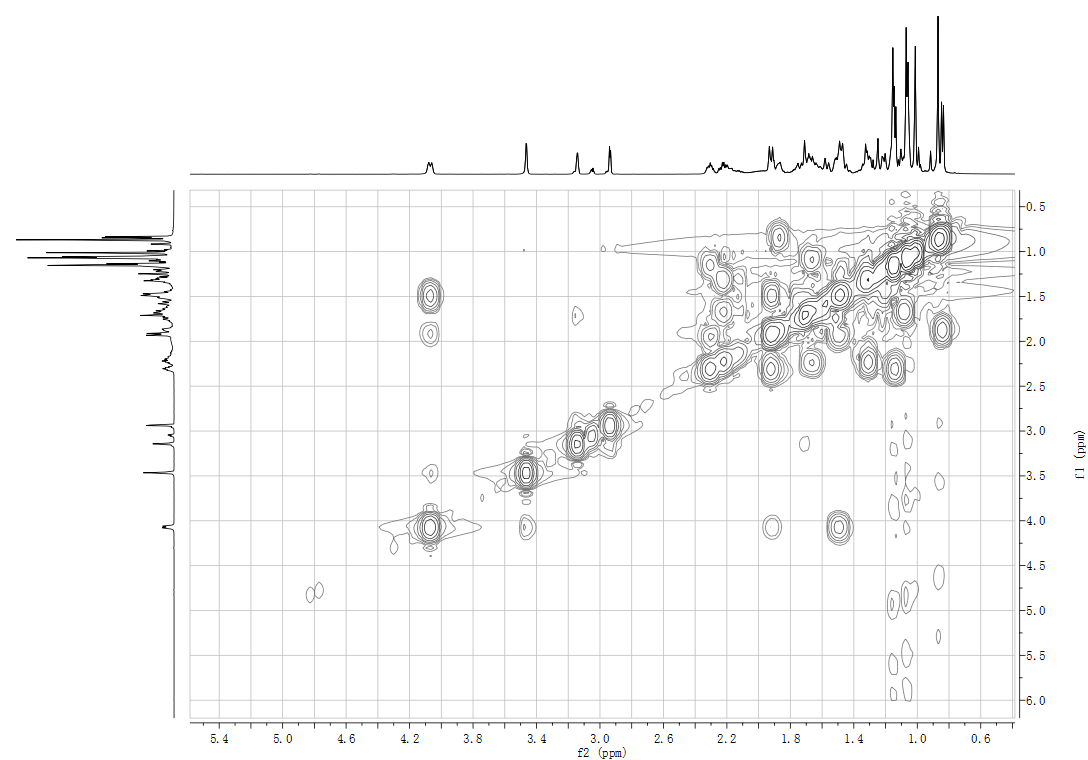
**

**Figure S11.** ^1^H-^1^H COSY spectrum of compound **2**.

**
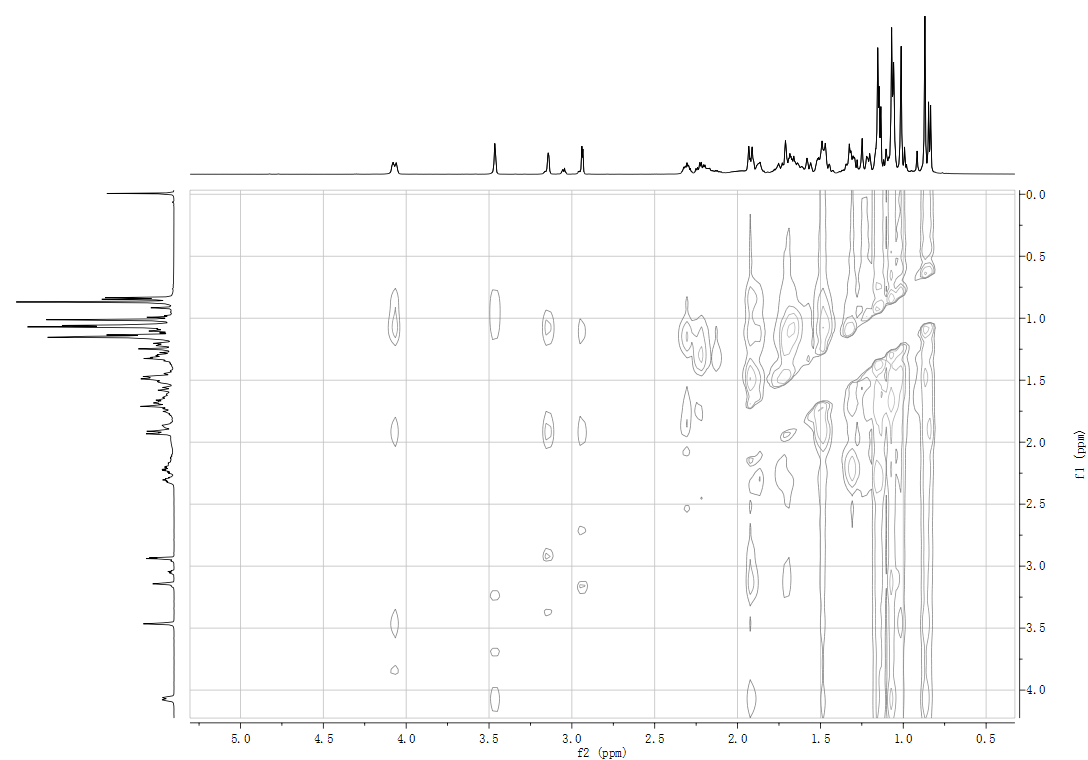
**

**Figure S12.** ROESY spectrum of compound **2**.
